# A Genome-Scale Metabolic Model of *Anabaena* 33047 to Guide Genetic Modifications to Overproduce Nylon Monomers

**DOI:** 10.3390/metabo11030168

**Published:** 2021-03-15

**Authors:** John I. Hendry, Hoang V. Dinh, Debolina Sarkar, Lin Wang, Anindita Bandyopadhyay, Himadri B. Pakrasi, Costas D. Maranas

**Affiliations:** 1 Department of Chemical Engineering, The Pennsylvania State University, University Park, State College, PA 16802, USA; jih11@psu.edu (J.I.H.); hvd5034@psu.edu (H.V.D.); dxs498@psu.edu (D.S.); lqw5322@psu.edu (L.W.); 2 Department of Biology, Washington University, St. Louis, MO 63130, USA; anindita@wustl.edu (A.B.); pakrasi@wustl.edu (H.B.P.)

**Keywords:** cyanobacteria, *Anabaena* sp. ATCC 33047, genome scale metabolic models, flux balance analysis, OptForce, caprolactam, valerolactam, heterocyst

## Abstract

Nitrogen fixing-cyanobacteria can significantly improve the economic feasibility of cyanobacterial production processes by eliminating the requirement for reduced nitrogen. *Anabaena* sp. ATCC 33047 is a marine, heterocyst forming, nitrogen fixing cyanobacteria with a very short doubling time of 3.8 h. We developed a comprehensive genome-scale metabolic (GSM) model, *i*AnC892, for this organism using annotations and content obtained from multiple databases. *i*AnC892 describes both the vegetative and heterocyst cell types found in the filaments of *Anabaena* sp. ATCC 33047. *i*AnC892 includes 953 unique reactions and accounts for the annotation of 892 genes. Comparison of *i*AnC892 reaction content with the GSM of *Anabaena* sp. PCC 7120 revealed that there are 109 reactions including uptake hydrogenase, pyruvate decarboxylase, and pyruvate-formate lyase unique to *i*AnC892. *i*AnC892 enabled the analysis of energy production pathways in the heterocyst by allowing the cell specific deactivation of light dependent electron transport chain and glucose-6-phosphate metabolizing pathways. The analysis revealed the importance of light dependent electron transport in generating ATP and NADPH at the required ratio for optimal N_2_ fixation. When used alongside the strain design algorithm, OptForce, *i*AnC892 recapitulated several of the experimentally successful genetic intervention strategies that over produced valerolactam and caprolactam precursors.

## 1. Introduction

Photosynthesis sustains almost all life on earth and offers an attractive route for chemical synthesis from H_2_O and CO_2_ using sunlight as the sole energy source. As production hosts, cyanobacteria have several advantages over eukaryotic plants and algae, including faster growth and higher photosynthetic efficiency [1,2]. They are also more amenable to genetic manipulations than their eukaryotic algal counterparts [3] and cyanobacteria-based production processes do not compete with food production for arable lands [1]. Studies have shown that cyanobacteria can be used to produce a wide variety of chemicals including alcohols, ketones, fatty acids, organic acids, and sugar feed stocks [3,4]. However, the production rates in these proof-of-concept studies are below industrially relevant targets. The recent discovery of a fast-growing cyanobacterium *Synechococcus elongatus* UTEX 2973 has provided us with an opportunity to overcome productivity barriers [5,6]. In addition to growth rate limitations, fresh water availability and nitrogen supplementation [7] are significant impediments for the success of cyanobacteria-based production processes. These hurdles can be overcome by utilizing a nitrogen fixing cyanobacteria that grows in sea water.

*Anabaena* sp. ATCC 33047 (refer to as *Anabaena* 33047) is a fast growing, filamentous, marine, nitrogen-fixing, heterocyst forming cyanobacteria. At a light intensity of 1500 μE m^−2^ s^−1^, it has a doubling time of 3.8 h, which is one of the shortest for a nitrogen-fixing cyanobacterium (Additional File S1). The organism is capable of growing at a wide range of temperature, pH, and salinity [8] consistent with large-scale industrial applications. It has been extensively studied for its potential for CO_2_ sequestration [9,10,11]. One such study reported a maximum CO_2_ fixation rate of 6.6 g L^−1^ day^−1^ [11,12], which is one of the highest for cyanobacteria. Such high CO_2_ fixation rate allude to the potential to achieve high titers of desired products and chemicals.

Recently, the genome sequence of this organism was published [13], opening up an opportunity to explore its metabolism using genome-scale metabolic (GSM) models. GSM models catalogue a system-level understanding of cellular metabolism using primarily reaction stoichiometry, biomass composition, and growth-associated maintenance/non-growth-associated maintenance estimates. Once a GSM is constructed, a variety of constraint-based reconstruction analysis methods can be used to obtain important information about metabolism. Flux balance analysis (FBA) enables calculation of theoretical maximum yield of biomass as well as of native and non-native metabolites of interest [14]. Flux variability analysis (FVA) [15] enables the determination of feasible flux ranges for the model reactions under a given condition. There are also a variety of strain design algorithms that facilitate the application of GSM models for metabolic engineering [16]. Indeed, genome scale models have been used to engineer diverse cyanobacteria towards the production of a variety of chemicals [16].

In this study, we developed a comprehensive GSM model (*i*AnC892) for the cyanobacterium *Anabaena* 33047 by pooling annotations from multiple sources. The two-cell model (*i*AnC892), consisting of vegetative cell and heterocyst, precisely captures the diazotrophic life cycle of this organism. Comparison with GSM of *Anabaena* sp. PCC 7120 (hereafter referred as model 7120) [17] revealed that there are 109 reactions that were present only in *i*AnC892 and not in model 7120. Analysis using *i*AnC892 revealed that the light dependent electron transport chain (LETC) is important for generating ATP and NA(P)DH in an appropriate ratio for optimal N_2_ fixation. Further, it predicted that glycolysis and TCA cycle can also act as a source of reducing equivalents for optimal N_2_ fixation. In order to demonstrate the predictive power of the model, it was deployed alongside the OptForce algorithm [18] to identify strain design strategies for overproducing valerolactam and caprolactam. The predictions captured many of the strategies reported in the literature for the overproduction of target chemicals or their precursors.

## 2. Results and Discussion

### 2.1. Genome Scale Metabolic Model of Anabaena 33047

**Figure 1:**
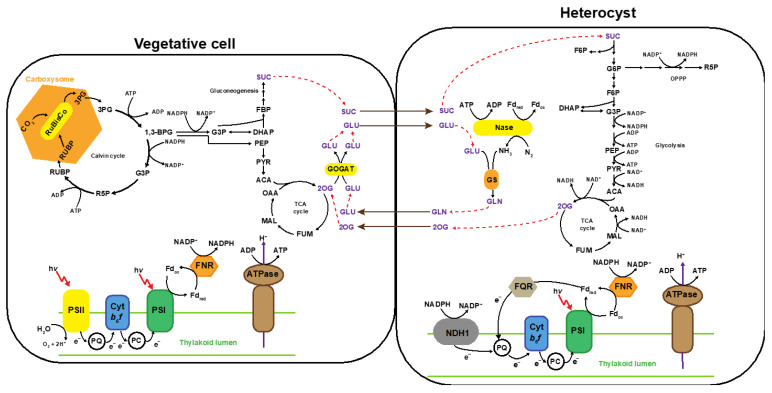
Two cell model of *Anabaena* 33047. The genome-scale metabolic model *i*AnC892 account for two super-compartments, the vegetative cell and the heterocyst, to capture the diazotrophic life style of this cyanobacterium. The two super-compartments differ only in the presence/absence of few modules colored in yellow. Red dotted lines trace the flow of exchange metabolites between the compartments. Abbreviations: 1,3-BPG, 1,3-bisphosphoglycerate; 3PG, 3-phosphoglycerate; ACA, acetyl-CoA; ATPase, ATP synthase; Cyt b_6_f, cytochrome b_6_f; DHAP, dihydroxyacetone phosphate; F6P, fructose-6-phosphate; FBP, fructose-1,6-bisphosphate; FNR, ferredoxin/NADP ^+^ reductase; Fd_ox_, oxidized ferredoxin; Fd_red_, reduced ferredoxin; FQR, ferredoxin/quinone reductase; FUM, fumarate; G3P, glyceraldehyde-3-phosphate; G6P, glucose-6-phosphate; GLN, glutamine; GLU, glutamate; GOGAT, glutamine oxoglutarate aminotransferase; MAL, malate; N_2_ase, nitrogenase; NDH1, NADPH dehydrogenase; OAA, oxaloacetate; OPPP, oxidative pentose phosphate pathway; PC, plastocyanin; PEP, phosphoenolpyruvate; PQ, plastoquinone; PSI, photosystem I, PYR, pyruvate; R5P, ribose-5-phosphate; RUBP, ribulose-1,5-bisphosphate; RuBisCo, ribulose 1,5-bisphosphate carboxylase/oxygenase; SUC, sucrose.

The GSM model (*i*AnC892) for the diazotrophic cyanobacterium *Anabaena* 33047 contains 953 unique reactions and 834 unique metabolites accounting for the annotation of 892 genes. Under nitrogen fixing condition, 8–9% [19] of cells in the filament of *Anabaena* 33047 become terminally differentiated into specialized cells called heterocysts. The heterocysts express nitrogenase (N_2_ase) enzyme and act as centers for nitrogen fixation. Because N_2_ase activity is inhibited by oxygen, heterocysts maintain low oxygen levels by dispensing oxygen generating photosystem II and developing a multilayered cell envelop that protects the enzyme from oxygen generated in neighboring vegetative cells [20,21]. Heterocysts lack both ribulose 1,5-bisphosphate carboxylase/oxygenase (RuBisCo) and carboxysome and therefore cannot fix CO_2_ [20,21]. In this manner, the incompatible processes of photosynthesis and nitrogen fixation are spatially separated in *Anabaena* 33047. In order to capture this division of labor, *i*AnC892 was constructed as a two-cell model (Figure 1), with two super-compartments: vegetative cell and heterocyst. The vegetative cell (906 reactions) and heterocyst (895 reactions) super-compartments have 892 reactions in common. They only differ in the presence/absence of a few but important metabolic processes. The heterocyst super-compartment lacks the photosystem II, RuBisCo and glutamine oxoglutarate aminotransferase [22] reactions. The vegetative cell super-compartment lacks the N_2_ase reaction.

The source of carbon and electron for the heterocyst is assumed to be sucrose, based on literature evidence [21,22,23,24]. Glutamate produced in vegetative cells, reportedly, acts as a carbon skeleton to incorporate ammonia produced from N_2_ fixation in the heterocyst [22]. Glutamine produced in the heterocyst was proposed to act as a nitrogen carrier [21,22,23,24,25,26]. The absence of glutamine oxoglutarate aminotransferase enzyme in the heterocyst results in the accumulation of 2-oxoglutarate, requiring its subsequent transport to vegetative cells [22,27]. Thus, in *i*AnC892, sucrose, glutamate, glutamine, and 2-oxoglutarate are allowed to exchange (non-facilitated) between vegetative cell and heterocyst compartments. Both the super-compartments were assumed to have the same biomass composition and a biomass formation equation was formulated and included in each of them. Although heterocysts do not undergo division, the corresponding compartment still included a biomass formation equation to act as a proxy for macromolecule turnover in this compartment. For FBA calculations, the biomass drain of vegetative cell is set as the objective function since only vegetative cells undergo cell division [17].

### 2.2. Comparison with the Metabolic Network of *Anabaena* sp. PCC 7120

**Figure 2:**
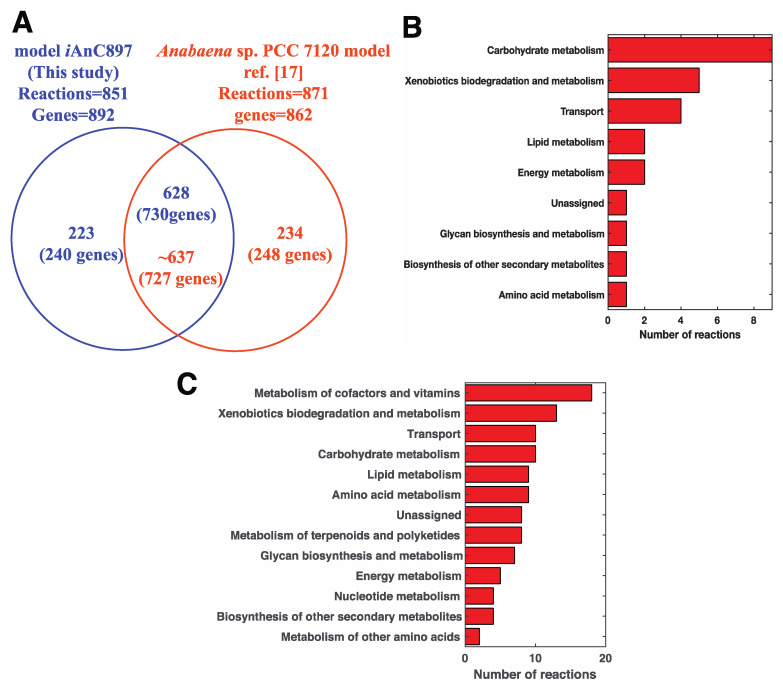
Comparison of the reaction content of *i*AnC892 and the recently published model for *Anabaena* sp. PCC 7120. (**A**) Venn diagram summarizing the results from the comparison of models. The exchange reactions, demand reactions, and non-facilitated transport reactions were excluded from this analysis. (**B**) A bar graph showing the distribution of reactions present only in *Anabaena* sp. ATCC 33047 and not in *Anabaena* sp. PCC 7120. For the genes associated with these reactions, BLAST bidirectional best hit did not identify any homologues in the genome of *Anabaena* sp. PCC 7120. (**C**) A bar graph showing the distribution of reactions reaction found to be present only in *i*AnC892 and not in *i*AM957.

After the model was constructed, it was benchmarked by comparing its contents with existing models. *i*AnC892 was first compared with the recently published GSM model for *Anabaena* sp. PCC 7120 (hereafter model 7120) [17]. Exchange reactions, demand reactions, and non-facilitated transport reactions were excluded from this analysis as these reactions are not associated with genes. Thus, out of the 953 reactions, only 851 were considered in this analysis. Out of the 851 reactions, *i*AnC892 shared 628 reactions with model 7120 (see Figure 2A) spanning 730 genes. The remaining 223 reactions, which were unique to *i*AnC892, were classified into four groups (Table S1). The first group contains 25 reactions whose associated genes had no homologues in the genome of *Anabaena* sp. PCC 7120 (*Anabaena* 7120). They belong to carbohydrate metabolism and xenobiotic degradation and metabolism (Figure 2B). The second group contains 84 reactions whose associated genes have homologues in the genome of *Anabaena* 7120 but they were not included in model 7120. The third group includes 38 reactions whose associated genes had homologues in model 7120 but the corresponding reactions are abstracted differently between the two models. The fourth group includes 76 reactions with GPRs identical to reactions in the shared-reaction set but different substrate and/or cofactor specificity compared to the corresponding reactions present in model 7120. Thus, only reactions in group 1 and group 2 are unique to *i*AnC892 since their genes are not represented in model 7120. These sets included reactions that can impact flux predictions under certain conditions. For example, pyruvate decarboxylase (EC 4.1.1.1) [28] and pyruvate formate-lyase (EC 2.3.1.54) [29] in group 1 are involved in the production of fermentative products and can impact predicted flux distribution under dark anoxic conditions. Similarly, uptake hydrogenase [30] in group II can impact the energetics in the heterocyst. The above analysis clearly indicates that *i*AnC892 is different from model 7120 in its reaction content and these differences have the potential to impact the flux distribution under certain conditions.

Similar comparison was also made with a model of *Anabaena Variabilis* ATCC 29413, *i*AM957 [31]. The analysis revealed 107 reactions falling in group 1 and group 2 categories mentioned above (Table S1). They included reactions in diverse metabolic subsystems as shown in Figure 2C including the primary ones such as amino acid metabolism, nucleotide metabolism, carbohydrate metabolism, and lipid metabolisms. Importantly, *i*AnC892 allowed for the synthesis of certain carotenoids such as adonixanthin, plectaniaxanthin etc., which were not made by *i*AM957. In addition, *i*AnC892 allowed the synthesis of myxoxanthophylls unlike *i*AM957. Further, *i*AnC892 included pyruvate formate-lyase (EC 2.3.1.54) which could potentially affect the flux distribution under dark anoxic conditions. These differences clearly indicate the uniqueness of *i*AnC892 in comparison to *i*AM957.

### 2.3. Flux Distribution Predicted by the Model

After benchmarking, *i*AnC892 was used to calculate flux ranges under diazotrophic conditions (Table S2) using FVA [15]. The bicarbonate uptake rate is constrained to the minimum value (8.5 mmol/gDW/h) required to achieve experimentally estimated growth rate (0.1824 h^−1^). The growth rate was constrained to experimental value (0.1824 h^−1^). Minimal fluxes required through key reactions under optimal conditions are shown in Table 1. According to FVA estimated flux ranges, the vegetative cell compartment of *i*AnC892 performed CO_2_ fixation with reducing equivalents and ATP replenished by LETC. Sucrose supplied by vegetative cell was used as electron source for heterocyst metabolism. The minimum sucrose transport rate to heterocyst indicates that at least 22% of carbon fixed in vegetative cells is metabolized in the heterocyst. N_2_ fixation was confined to heterocyst. The minimum N_2_ase flux required to sustain optimal growth rate was found to be 2.5 fold lower than the N_2_ase activity measured for this organism, 1.7 mmol/gDW/h, using acetylene reduction assay [32]. This 2.5-fold difference is comparable to the 3-fold difference assumed to exist between the nitrogen fixation activity and acetylene reduction activity of N_2_ase [33]. This would indicate that the available N_2_ase flux capacity is close to the minimal value required for the optimal growth. The minimum flux required by heterocyst-PSI indicated that LETC is used for ATP synthesis in the heterocyst. The ammonia produced as a product of N_2_ fixation was incorporated into glutamate supplied by vegetative cell, through glutamine synthetase reaction, to form glutamine. The glutamine formed is then transported back to vegetative cell as a nitrogen carrier. The above pathway utilization pattern was found to be qualitatively similar to what was reported using GSM models for *Anabaena* sp. PCC 7120 [17] and *Anabaena Variabilis* ATCC 29413 [31]. Thus, the pathway usage predicted by *i*AnC892 agrees with the current understanding of metabolism of heterocystous cyanobacteria.

### 2.4. ATP/NAD(P)H Sources for N_2_ Fixation in Heterocyst

The N_2_ fixation by N_2_ase has a high energy demand, requiring 16 ATP and 4 NAD(P)H or 8e^−^ for every one mole of N_2_ fixed [34]. In the heterocyst, substrate level phosphorylation in glycolysis, LETC, and respiratory electron transport chain (RETC) can generate the required ATP. The glycolysis, pentose phosphate pathway (PPP) and tricarboxylic acid (TCA) cycle can provide the reducing equivalents. Experimental observations have indicated LETC and PPP as the major contributors of ATP and reducing equivalents respectively for N_2_ synthesis in heterocysts [35,36,37]. However, the relative importance of other pathways and to what extent they can support growth individually are not yet clearly understood. Since *i*AnC892 allows for cell selective activation or inactivation of pathways, it offers an opportunity to study these pathways individually. For this analysis, it was assumed that there is no macromolecular turnover in the heterocyst. This makes N_2_ fixation reactions, N_2_ase and glutamine synthetase, as the only two energy demanding reactions in the heterocyst. Since, under diazotrophic conditions, optimal growth rate requires a minimum N_2_ fixation rate, growth rate was chosen as an indirect metric to evaluate the different ATP/NAD(P)H synthesis routes in the heterocyst. Additional metrics include the minimum sucrose uptake rate and minimum CO_2_ evolution rate in the heterocyst. Higher sucrose demand might indicate poor energy efficiency of the pathway under consideration. Higher CO_2_ evolution indicates poor carbon efficiency and the poor gas exchange property of the heterocyst membrane [38] might limit the pathway flux.

**Figure 3:**
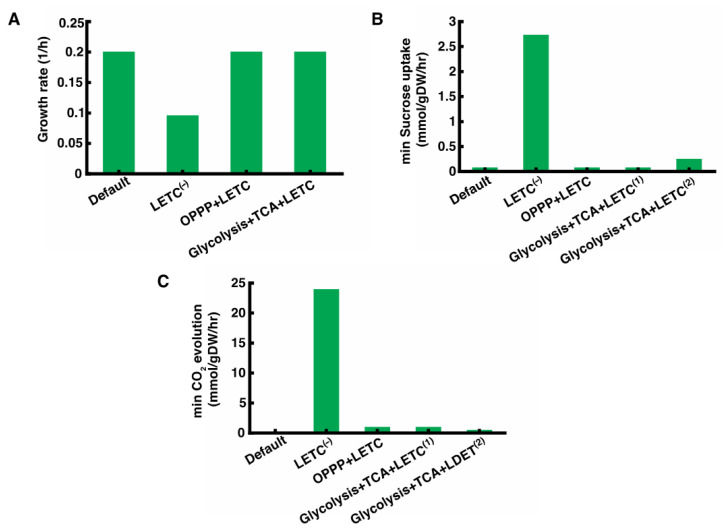
Evaluation of ATP/NADPH generating schemes in the heterocyst. (**A**) Bar graph showing the maximum growth rate supported by different ATP/NADPH generating schemes in the heterocyst. (**B**) Bar graph showing minimum sucrose uptake flux required by heterocyst while utilizing the different schemes. (**C**) Bar graph showing the minimum CO_2_ evolution rate associated with various schemes. The default model utilizes the unmodified metabolic network in the heterocyst.

First, in order to understand the importance of LETC, the ability of the heterocyst metabolic network to sustain N_2_ fixation without LETC was evaluated. The LETC was inactivated in *i*AnC892 by blocking light uptake (Section 3.3). Under LETC^(−)^ condition, *i*AnC892 predicted a 2-fold decrease in growth rate (Figure 3A). This is primarily because of the loss of 50% of fixed carbon due to secretion of ethanol. The main reason behind this is that this metabolic mode produces ATP and NADPH in a fixed ratio that did not match the ratio required by N_2_ fixation (4:1). For example, the ATP/NAD(P)H ratio calculated using pFBA solution is 1:8 which does not match the ratio of 4:1 required for N_2_ fixation. Due to this mismatch, the excess reducing equivalents had to be siphoned off (e.g., H_2_ production) or converted to ATP using RETC. Unfortunately, this was not sufficiently facilitated by RETC, due to oxygen uptake constraint (Section 3.2), or H_2_ production, and hence there is a need to utilize the fermentative pathways. This clearly highlights the necessity of generating ATP and NAD(P)H in the appropriate ratio for optimal N_2_ fixation in the heterocyst. Additionally, it highlights the importance of retaining PSI in the heterocyst as it can modulate the ATP/NAD(P)H ratio [39], through cyclic electron transport, to meet the N_2_ fixation demand.

The most commonly suggested scheme of ATP and NAD(P)H synthesis for N_2_ fixation uses pentose phosphate pathway (PPP) and LETC respectively [34,40]. In the PPP + LETC scheme, NADPH is supplied by PPP while ATP is supplied by LETC. The cyclic electron flow around PSI in LETC can be used to modulate ATP yield per electron, and in turn, the ATP:NADPH ratio to match the value specified by N_2_ fixation. Thus, this scheme decouples ATP synthesis from NADPH synthesis. Operation of this metabolic mode in the heterocyst is experimentally confirmed by the loss of N_2_ fixation in a *zwf* mutant of *Nostoc* sp. ATCC 29133 [37]. *i*AnC892 was used to evaluate this scheme by knocking out reactions that would allow glucose-6-phosphate (G6P) and PPP intermediates (glyceraldehyde-3-phosphate, fructose-6-phosphate) to enter into glycolysis. Under this condition, *i*AnC892 predicted growth rate was equal to optimal growth rate (Figure 3A). The minimum sucrose flux required under this condition was equal to the minimum value required by the unaltered model (Figure 3B). This indicates that this metabolic mode does not impose an increased demand of sucrose supply. However, the complete oxidation of G6P to CO_2_ through PPP resulted in CO_2_ evolution from heterocyst (Figure 3C). Thus, utilization of this pathway might have poor carbon conversion efficiency unless the liberated CO_2_ is re-fixed by vegetative cells.

Another plausible scheme uses glycolysis and the TCA cycle to metabolize the G6P generated from sucrose. In this scheme, glycolysis and the TCA cycle provide ATP and NADPH in a fixed ratio which is in turn modulated by the LETC-associated ATP synthesis to match the demand of N_2_ fixation. The evidence for operation of this mode (glycolysis + TCA + LETC) comes from the observations that N_2_ase activity is stimulated in cell free extracts of the heterocyst by intermediates of glycolysis and the TCA cycle [41,42]. The glycolysis + TCA + LETC scheme was also evaluated using *i*AnC892 by allowing G6P to be metabolized only through glycolysis and the TCA cycle (Section 3.3). It was observed that this scheme can result in either complete or incomplete oxidation of G6P and achieving optimal growth in both the cases (Figure 3A). The complete oxidation of G6P into CO_2_ was facilitated by a complete TCA cycle. Under this condition (glycolysis + TCA + LETC^(1)^), the predicted minimum rate of CO_2_ evolution and minimum sucrose uptake by heterocyst were equal to that observed in the PPP + LETC scheme (Figure 3B,C). Alternatively, the TCA cycle can be operated in an incomplete format where both 2-oxoglutarate and CO_2_ are formed as end products of G6P metabolism. The 2-oxoglutarate is then transported to vegetative cell, where it is further metabolized. Under this condition (glycolysis + TCA + LETC^(2)^), the minimum rate of CO_2_ evolution from heterocyst is 2-fold lesser than what was observed in the PPP + LETC scheme (Figure 3C). Thus, this mode provides a better carbon efficiency compared to the PPP + LETC scheme. However, the minimum sucrose uptake rate increased by 3-fold compared to the PPP + LETC scheme (Figure 3B).

The above analysis clearly indicates that LETC, with its cyclic electron flow, is important to generate ATP and NADPH in the ratio required for nitrogen fixation without compensating on growth rate. In this role, it can be coupled with different G6P metabolizing routes in the heterocyst resulting in optimal nitrogen fixation and growth rate. In addition to the most commonly suggested PPP + LETC scheme, glycolysis + TCA cycle + LETC scheme was also found to support optimal N_2_ fixation rates with similar sucrose demand and carbon conversion. Studies with cell free extracts has suggested that TCA cycle can proceed up to synthesis of 2-oxoglutarate in the heterocyst [41,42]. Since the existence of a complete TCA cycle in cyanobacteria has been confirmed [43], it cannot be ruled out that glycolysis + TCA cycle + LETC also contributes in supplying the reducing equivalents for N_2_ fixation.

### 2.5. Strain Designs for Caprolactam and Valerolactam Overproduction

**Figure 4:**
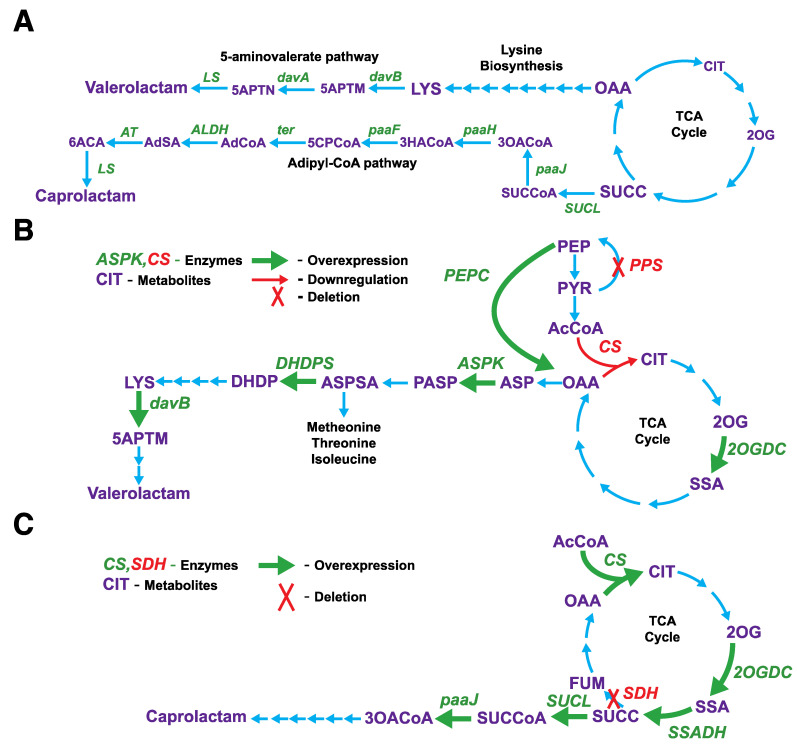
OptForce-based prediction of intervention strategies for the overproduction of valerolactam and caprolactam in *Anabaena* 33047 using *i*AnC892. (**A**) Schematic of pathways used for the in-silico production of valerolactam and caprolactam in *i*AnC892. (**B**) Schematic depicting the intervention strategies identified by OptForce to enable overproduction of valerolactam in *Anabaena* 33047. (**C**) Schematic depicting the intervention strategies identified by OptForce to enable overproduction of caprolactam in *Anabaena* 33047. Abbreviation: 2OGDC, 2-oxoglutarate decarboxylase; 3HACoA, 3-hydroxyadipyl CoA; 3OACoA, 3-oxoadipyl CoA; 5APTM, 5-aminopentanamide; 5APTN, 5-aminopentanoate; 5CPCoA, 5-carboxy-2-pentenoyl-CoA; 6ACA, 6-aminocaproic_acid; AdCoA, adipyl-CoA; AdSA, adipate-semialdehyde; ALDH, aldehyde dehydrogenase; ASPK, aspartate kinase; ASPSA, aspartate4-semialdehyde; Asp, aspartate; AT, aminotransferase; CS, citrate synthase; DHDP, dihydrodipicolinate; DHDPS, dihydropicolinate synthetase; davA, 5-aminopentanamidase; davB, lysine 2-monooxygenase; LS, lactam synthase; LYS, lysine; PASP, 4-phospho-L-aspartate; PEPC, phosphoenolpyruvate carboxylase; PPS, phosphopyruvate synthetase; paaF, 2,3-dehydroadipyl-CoA hydratase; paaH, 3-hydroxyacyl-CoA reductase; paaJ, 3-oxoadipyl-CoA thiolase; SDH, succinate dehydrogenase; SSADH, succinate-semialdehyde dehydrogenase; SUCC, succinate; SUCCoA, succinyl CoA; SUCL, succinate-CoA ligase; ter, trans-enoyl-CoA reductase;.

One of the applications of *i*AnC892 is to predict genetic interventions that lead to the over production of target chemicals in *Anabaena* 33047. In order to demonstrate this capability, we used it alongside the OptForce [18] algorithm to predict metabolic engineering strategies that overproduce valerolactam and caprolactam. The production of valerolactam and caprolactam were enabled by adding the 5-aminovalerate pathway [44] and adipyl-CoA pathway [45], respectively, to *i*AnC892 (Figure 4A).

#### 2.5.1. Interventions for the Overproduction for Valerolactam

In the case of valerolactam production, comparison of flux ranges of wild type and overproduction strains identified 18 upregulation, 6 downregulation, and 0 knockout interventions. The OptForce algorithm was then used to identify combinations of these interventions that would result in the overproduction of valerolactam. The strategies in the current study are summarized in Table 2 and Figure 4B depicts the associated interventions in a pathway schematic. Initially, three single intervention strategies were predicted involving the upregulation of aspartate kinase, dihydrodipicolinate synthase, and lysine 2-monooxygenase (Mutants 1–3 in Table 2). Aspartate kinase upregulation is intended to push aspartate towards lysine synthesis. Indeed, upregulation of native aspartate kinase has been shown to significantly improve (2-fold) lysine production in *B. Methanolicus* [46]. Dihydrodipicolinate synthase overexpression serves to draw aspartate semialdehyde away from synthesis of other amino acids and direct it towards lysine synthesis (Figure 4B). Stand-alone overexpression of dihydrodipicolinate synthase has been shown to be enough to convert wild-type *C. glutamicum* into a lysine producing strain demonstrating its usefulness in redirecting flux towards lysine production [47]. In this analysis, lysine 2-monooxygenase is included as a proxy for the 5-aminovalerate pathway (Figure 4A) and its overexpression implies enhancing the flux through this pathway. Indeed production levels of valerolactam precursor 5-aminopentanoate has been shown to increase with the expression levels of this pathway enzymes in *C. glutamicum* [48].

To identify other suboptimal strategies, OptForce analysis was repeated after removing the above three candidates from the MUST^U^ set. However, the algorithm did not predict any overproduction strategies in this second round. Close examination revealed the existence of a futile cycle involving potential overexpression candidates and rendering them ineffective. The discovered cycle was removed by implementing phosphoenolpyruvate synthase knockout in the model and the algorithm was then able to predict few more strategies (Mutants 4–6 in Table 2). All newly predicted strategies involved downregulation of citrate synthase as a means of increasing the availability of key precursor, oxaloacetate, for lysine synthesis. The citrate synthase downregulation was included as a proxy for the downregulation of coupled reactions, citrate synthase, aconitase, and isocitrate dehydrogenase in *i*AnC892. Reduced activity of citrate synthase [49] or downregulation of isocitrate dehydrogenase [50] has been shown to enhance the production of lysine in *C. glutamicum*. In *i*AnC892, down regulation of citrate synthase has to be coupled with over expression of phosphoenolpyruvate carboxylase (Mutant 4 in Table 2) or 2-oxoglutarate dehydrogenase (Mutant 5 in Table 2) to push the flux towards the aspartate pathway via increased oxaloacetate availability. Of these two overexpression candidates, phosphoenolpyruvate carboxylase has been shown to enhance the production of valerolactam in *E. coli* [51]. Combining the downregulation of citrate synthase with overexpression of phosphoenolpyruvate carboxylase and 2-oxoglutarate dehydrogenase (Mutant 6 in Table 2) in *i*AnC892, had a synergetic effect that increased the production yield of valerolactam by 5-fold compared to overexpression of only one of the two (Mutant 4 and 5 in Table 2).

#### 2.5.2. Interventions for the Overproduction for Caprolactam

In case of caprolactam production, flux range comparison between wild type and overproduction strain identified 17 upregulation, 2 downregulation, and 0 knockout interventions. Literature search revealed several studies where knockout of succinate dehydrogenase had contributed to the overproduction of caprolactam precursors adipic acid [52] and succinate [53,54,55]. Therefore, succinate dehydrogenase was manually included as a knockout candidate during OptForce analysis. The intervention strategies identified from our analysis are summarized in Table 3 and Figure 4C contextualizes these interventions on a metabolic pathway schematic. Initially, the OptForce algorithm predicted two strategies each consisting of a single intervention, upregulation of 3-oxoadipyl-CoA thiolase (Mutant 1 in Table 3) and upregulation of succinate-CoA ligase (Mutant 2 in Table 3). In this analysis, 3-oxoadipyl-CoA thiolase is a proxy for the entire caprolactam biosynthesis pathway as it is linear. Thus, this strategy in reality alludes to facilitation of an overall increase in the flux through this pathway. Effectiveness of this strategy has been experimentally demonstrated both by elimination of pathway bottle necks [56] and recruiting suitable enzyme combinations [52] in *E. coli*. Overexpression of succinate-CoA ligase seeks to increase the availability of succinyl-CoA, substrate for the adipyl-CoA pathway. Cyanobacterial TCA cycle has succinate as an intermediate instead of succinyl-CoA (Figure 4A) [43]. Therefore, succinyl-CoA has to be produced from succinate by succinate:CoA ligase (Figure 4A). Hence, this strategy is unique to cyanobacteria. In contrast, down regulation of succinate-CoA ligase is needed for increasing the availability of succinyl-CoA in organisms with the classical TCA cycle [56].

To find alternate strategies, OptForce analysis was repeated after removing 3-oxoadipyl-CoA thiolase and succinate-CoA ligase from MUST^U^ set. All newly identified strategies involved the knockout of succinate dehydrogenase. This strategy is intended to increase succinate availability for caprolactam synthesis by preventing its conversion to fumarate. This intervention was combined with the over expression of one of the following three enzymes in the identified strategies: 2-oxoglutarate decarboxylase, citrate synthase, and succinate semialdehyde dehydrogenase; all of them push the flux towards the synthesis of succinate. Over expression of 2-oxoglutarate decarboxylase and succinate semialdehyde dehydrogenase had been shown to increase succinate production in *Synechococcus elongatus* PCC 7942 [57]. Over expression of citrate synthase and knockdown of succinate dehydrogenase has been shown to enhance the production of succinate in *Synechococcus elongatus* PCC 11801 [55].

## 3. Materials and Methods

### 3.1. Genome Scale Metabolic Model

The genome sequence of *Anabaena* 33047 was obtained from the NCBI database. Metabolic function annotations for individual genes were obtained from multiple pathway databases using BLAST homology searches. Biochemical functions from Metacyc [58], KEGG [59], and published model of *Anabaena* sp. PCC 7120 [17] were obtained using the Raven Toolbox [60]. Additional annotations were also obtained from the automated annotation pipeline RAST [61]. Annotations in the form of EC numbers obtained from the above databases were juxtaposed against each other to assign metabolic functions to as many genes as possible. The reactions corresponding to these biochemical functions were first retrieved from curated cyanobacterial models and if not available in these models, then retrieved from the database model SEED [62]. The metabolic network reconstruction was then gap filled for the production of biosynthetic precursors under photoautotrophic condition. The metabolic reconstruction was then manually curated and the individual reactions were balanced for mass and charge. Thermodynamically infeasible loops were identified and removed. The two-cell model was then constructed out of the curated reactions based on the information obtained from literature and the previously published model for *Anabaena* sp. PCC 7120 [17]. For the formulation of biomass formation reaction, information about the amount of proteins, carbohydrates, lipids, RNA, and DNA in 1g biomass of *Anabaena* 33047 was obtained from literature sources [63]. The content of pigments (carotenoids and chlorophyl), peptidoglycan, lipopolysaccharide, soluble pool, and inorganic ions were assumed to be the same as in *Synechocystis* sp. PCC 6803 and the corresponding information was taken from the GSM model *i*JN678 [64]. The values obtained were rescaled to a total biomass of 1 g. The rescaled values were then used to obtain the stoichiometric coefficients of the biomass precursors in the biomass formation equation. The model consistency and quality were assessed using the metabolic model test (MEMOTE) suite [65] and the model was modified to fix the detected issues. The MEMOTE total score for the model is 76% with the model consistency score being 99% (Additional File S2). The model is available in Excel format (Additional File S3) and COBRA Toolbox compatible SBML (Additional File S4) and MATLAB (Additional File S5) format. A schematic representation of important pathways in the vegetative cell (Figure S1) and heterocyst compartment (Figure S2) of the model was constructed using Escher toolbox [66] and provided with the supplementary information.

### 3.2. Model Simulation under Diazotrophic Condition

FBA was used to query the model and to predict flux distributions under maximum biomass formation conditions [14]. All simulations were performed under carbon limited conditions with CO_2_ uptake restricted to an upper bound of 8.5 mmol/gDW/h. This value corresponds to the minimum CO_2_ uptake rate required to achieve the experimentally estimated growth rate of 0.1824 h^−1^. The upper bounds of PSI and PSII photon uptake were set to 100 mmol/gDW/h to restrict futile cycles but without limiting biomass yield. The upper bound on N_2_ uptake was set to a non- limiting value of 10 mmol/gDW/h. This value limits the potential futile cycle formed by N_2_ fixation and ammonia secretion. The flow of sucrose from vegetative cell to heterocyst and the flow of 2-oxoglutarate from heterocyst to vegetative cell were unconstrained. The glutamate–glutamine exchange between compartments was constrained so as the entire amount of glutamate from vegetative cell was converted to glutamine in the heterocyst. This prevents the heterocyst cell from using glutamate as a carbon source. Since only the vegetative cell undergoes cell division in the *Anabaena* 33047 filament, the biomass drain of the vegetative cell is set as the objective function for FBA calculations [17]. Even though the heterocyst cell does not undergo cell division, we set the lower bound of its biomass formation reaction to 10% of the experimentally estimated growth rate (i.e., 0.1 × 0.1824 h^−1^). This properly accounts for biomass turnover [17]. The oxygen uptake rate for heterocyst cultures of *Anabaena* 33047 in air saturated suspension was estimated to be 0.7 mmol/gDW/h [32]. Using this estimate and assuming that in a diazotrophic culture 9% of the cells are heterocysts, the upper bound on oxygen uptake by the heterocyst compartment in *i*AnC892 was calculated to be 0.063 mmol/gDW/h. Unless specified, simulations were performed with the above constraints. The identification of reaction flux ranges was carried out using FVA [15]. The COBRA Toolbox [67] running on MATLAB platform was used for both FBA and FVA simulations.

### 3.3. Analysis of ATP and NAD(P)H Sources in Heterocyst

Flux ranges of different metabolic schemes (Table S3) considered were obtained using FVA after constraining biomass flux to the maximum value obtained under respective conditions. LETC^(−)^ condition is simulated by setting the heterocyst light uptake flux to zero. OPPP + LETC condition was simulated by preventing G6P and PPP intermediates from entering into glycolysis. This is achieved by setting the upper bound of glucose-6-phosphate isomerase (*rxn00558__hc*) to zero to prevent G6P to F6P conversion. In addition, flux through phosphoketolase (*rxn01187__hc* and *rxn00548__hc*) and glyceraldehyde-3-phosphate dehydrogenase (*rxn00781__hc*) were set to zero to prevent the PPP intermediates from entering into glycolysis. Glycolysis + TCA + LETC condition was simulated by setting the flux through glucose-6-phosphate dehydrogenase (*rxn00604__hc*) and phosphoketolase (*rxn01187__hc* and *rxn00548__hc*) reactions to zero. Flux ranges of glycolysis + TCA + LETC^(1)^ and glycolysis + TCA + LETC^(2)^ were obtained by additionally constraining the 2-oxoglutarate transport to vegetative cell (*akg_vc_hc_exch*) and heterocyst CO_2_ evolution rate (*cpd00011exccc__hc*) respectively. The flux ranges of glycolysis + TCA + LETC^(1)^ were obtained by setting the upper bound of 2-oxoglutarate transport to vegetative cell to the minimum value obtained for glycolysis + TCA + LETC condition. The flux ranges of glycolysis + TCA + LETC^(2)^ were obtained by constraining the heterocyst CO_2_ evolution rate to the minimum value obtained for glycolysis + TCA + LETC condition.

### 3.4. Strain Design Using OptForce

Details on optimization formulation and various steps involved in implementing the OptForce algorithm for a stoichiometric model can be found in [18] and [68]. The first step in the OptForce procedure is enumeration of flux ranges of wild-type strain. This is usually accomplished by constraining the flux ranges in GSM model using ^13^C-MFA derived flux ranges and then performing FVA (Table S4). Unfortunately, metabolic flux distribution of neither *Anabaena* 33047 nor any other heterocyst forming cyanobacteria is available. ^13^C-MFA-based intracellular flux estimates are available only for a few non-heterocystous cyanobacteria [69]. Among these, only *Synechococcus* 7002 occupies a marine habitat as *Anabaena* 33047 [70]. Therefore, flux distribution of this organism [71] was used as a proxy for wild-type metabolic flux distribution of *Anabaena* 33047. Measured flux ranges available for central carbon metabolism were directly mapped to vegetative cell compartment in *i*AnC892, since CO_2_ fixation is confined to this compartment. The imported flux ranges were then used to constrain the remaining reactions, including those in the heterocyst, using FVA. The second step is calculation of flux ranges for overproduction strain. To achieve this, the lower bound of product secretion is set to 90% of theoretical yield and the lower bound of biomass synthesis reaction is set to 10% of max biomass yield. With above constraints in place, FVA is now performed to identify the flux ranges of overproducing strain (Table S4). The flux ranges of overproducing strain and wild-type strain are contrasted against each other to identify the members of Force sets MUST^X^, MUST^U^, and MUST^L^. Additionally, the ranges of flux sums and flux differences were also compared to identify candidates of MUST^LL^, MUST^UU^ MUST^LU^, and MUST^UL^. Transport, exchange, demand, spontaneous and generalized reactions were removed from all MUST sets. Where the reactions were part of a linear sequence, only the first reaction is considered as perturbation candidate since all reactions in a linear pathway are coupled to each other. In vivo essential reactions and reactions carrying very low fluxes were removed from MUST^X^ sets resulting in empty MUST^X^ sets for both the products. Growth coupled reactions were excluded from the MUST^L^ set since strategies involving these will compromise on growth to overproduce the product. The force sets used to identify metabolic engineering strategies for overproduction of caprolactam and valerolactam are provided with the supplementary information (Table S5). Using a bi-level mixed-integer optimization formulation, perturbation strategies combining overexpression (MUST^U^ set), downregulation (MUST^L^ set), and knockout (MUST^X^ set) of reactions were identified for the overproduction of target chemicals. The OptForce simulations were carried out in GAMS (version 24.8.5 GAMS Development Corporation, Fairfax, VA, USA) environment with IBM ILOG CPLEX solver (version 12.7.1.0 IBM, New York, NY, USA) using the Roar supercomputer of Institute for Computational and Data Sciences, Pennsylvania State University, State College, PA, USA.

## 4. Conclusions

In this study we constructed a comprehensive GSM model, *i*AnC892, for the fast-growing, N_2_-fixing cyanobacteria, *Anabaena* 33047 by pooling together annotation information from diverse databases. Because *Anabaena* 33047 forms heterocysts under diazotrophic conditions, the *i*AnC892 model featured two super-compartments: vegetative cell and heterocyst. *i*AnC892 was able to accurately capture several aspects of the diazotrophic metabolism of heterocyst forming cyanobacteria. Reaction content comparison with *Anabaena* 7120 and *Anabaena* 29413 indicated that *i*AnC892 is different from these models in terms of its reaction content. Cell specific shutdown of heterocyst-LETC revealed its importance in generating ATP and NADPH at ratios optimal for N_2_ fixation. Similar analysis of heterocyst central carbon metabolism revealed the existence of alternative routes other than PPP that can supply reducing equivalents for N_2_ fixation. The usefulness of *i*AnC892 was further tested by using it alongside OptForce to predict genetic interventions for overproduction of valerolactam and caprolactam. The study recapitulated several of the experimentally successful strategies. Further improvement in predictions can be achieved by using flux distributions specifically generated for *Anabaena* 33047. Availability of species-specific flux distribution would also help pin down exchanges between vegetative cell and heterocysts which would provide a clearer picture of the energy production pathways active in the heterocyst.

## Figures and Tables

**Table 1:** Table showing model predicted minimum flux required through reactions of interest for optimal growth.

S.No	Reaction	Minimum Flux for Optimal Growth (mmol/gDW/h)
1	RuBisCo	9.5
2	Photosystem II	20.7
3	Thylakoid ATP synthase in vegetative cell	10.3
4	Sucrose uptake by heterocyst	0.15
5	N_2_ase	0.68
6	Photosystem I in heterocyst	15.6
7	Glutamate/glutamine exchange flux	1.2

**Table 2:** Genetic interventions suggested by OptForce for the overproduction of valerolactam. *k* defines the number of interventions allowed in the OptForce. The numerical values indicate the required fold change of overexpression/downregulation. Fold change overexpression is calculated by dividing OptForce predicted lower bounds for overproduction strains with the metabolic flux analysis (MFA) constrained upper bounds of wild type. Fold change downregulation was calculated by dividing the MFA constrained lower bounds of wild type with the OptForce predicted upper bounds for overproducing strains. ^a^ ↑ indicates overexpression. ↓ indicates downregulation. Δ indicates deletion. ^b^ These interventions are identified after applying integer cut sets to avoid FORCE sets corresponding to the strains #1–3. ^c^ Phosphoenolpyruvate synthetase knock out was applied before obtaining the FORCE sets for strains #4–6.

S. No	Interventions ^a^	Mutants					
		*k* = 1			*k* = *2*		*k* = *3*
		1	2	3	4 ^b^	5 ^b^	6 ^b^
1.	↑2-oxoglutarate decarboxylase					↑	↑
2.	↑Aspartate Kinase		1.2↑				
3.	↓Citrate Synthase				10↓	10↓	10↓
4.	↑Dihydrodipicolinate synthase			51↑			
5.	↑Lysine 2-monooxygenase	↑					
6.	↑Phosphoenolpyruvate Carboxylase				1.5↑		1.5↑
7.	ΔPhosphoenolpyruvate Synthetase ^c^				Δ	Δ	Δ
Valerolactam production flux (mmol/gDW/h)		1.25	1.25	1.25	0.23	0.23	1.18

**Table 3:** Genetic interventions identified by OptForce for overproduction of caprolactam. *k* defines the number of interventions allowed in the OptForce. The numerical values indicate the required fold change of overexpression obtained by dividing the OptForce predicted lower bounds for overproducing strain with the MFA constrained upper bound for the wild type. ^a^ ↑ indicates overexpression. Δ indicates deletion. ^b^ These interventions are identified after applying integer cut sets to avoid FORCE sets corresponding to the strains #1&2.

S. No	Interventions ^a^	Mutants				
		*k* = 1		*k* = 2	
		1	2	3 ^b^	4 ^b^	5 ^b^
1.	↑2-oxoglutarate decarboxylase				↑	
2.	↑3-oxoadipyl-CoA thiolase	↑				
3.	↑Citrate Synthase					6.3↑
4.	↑Succinate-CoA ligase		↑			
5.	↑Succinate-semialdehyde dehydrogenase			↑		
6.	ΔSuccinate dehydrogenase			Δ	Δ	Δ
Caprolactam production flux (mmol/gDW/h)		1.04	1.04	1.04	1.04	1.04

## Data Availability

The datasets supporting the conclusions of this article are included within the article and its additional files.

## Supplementary Materials

The following are available online at https://www.mdpi.com/2218-1989/11/3/168/s1, Figure S1: Schematic showing the important pathways in the Vegetative cell compartment of iAnC892, Figure S2: Schematic showing the important pathways in the heterocyst compartment of iAnC892, Table S1: Reaction in different categories identified in the comparison of iAnC892 with model 7120 and iAM957, Table S2: FVA estimated flux ranges for iAnC892 under nitrogen fixing, photoautotrophic condition, Table S3: Flux ranges corresponding to different ATP/NAD(P)H producing schemes in the heterocyst as calculated using FVA, Table S4: Flux ranges of wild type and overproducing strains as calculated for OptForce based strain design, Table S5: Spread sheet containing the force sets used to identify metabolic engineering strategies for overproduction of caprolactam and valerolactam, Additional File S1: Appendix text containing the materials and methods section for the growth experiments done on Anabaena 33047 and the associated results relevant for this study, Additional File S2: MEMOTE report for iAnC892, Additional File S3: The genome scale metabolic model of *Anabaena* sp. ATCC 33047, iAnC892, in Excel format, Additional File S4: The genome scale metabolic model of *Anabaena* sp. ATCC 33047, iAnC892, in the COBRA compatible SBML format, Additional File S5: The genome scale metabolic model of *Anabaena* sp. ATCC 33047, iAnC892, in the COBRA compatible MATLAB (.mat) format.
