# Supplementary material for: A Genome-Scale Metabolic Model of Anabaena 33047 to Guide Genetic Modifications to Overproduce Nylon Monomers
Source: Metabolites. 2021 Mar 15;11(3):168. doi: 10.3390/metabo11030168 (PMC7999273; doi:10.3390/metabo11030168)
Supplement: Supplementary file 1 [file metabolites-11-00168-s001.zip › supp_info/Additional File S1.docx]

**Appendix S1**

**Growth studies on *Anabaena* sp. ATCC 33047**

**Material and Method:** *Anabaena* sp. ATCC 33047 cells were grown in ASP2 medium lacking any source of fixed nitrogen in a Multi-Cultivator MC 1000-OD device (Photon Systems Instruments, Drasov, Czech Republic) under continuous light (LED light source). Cells were grown at 42°C in ambient air supplemented with 3% CO_2_ under light intensities 500 and 1500 μmol photons m^-2^s^-1^. Growth was monitored by measuring optical density at 730nm.

**Results and Discussion**


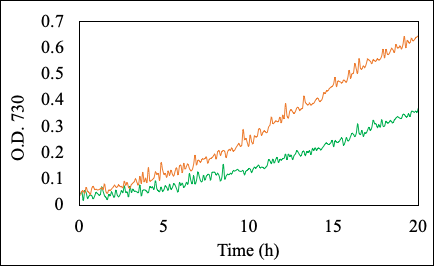


**Figure S1:** Growth of Anabaena 33047 under different light intensities.

The growth of *Anabaena* sp. ATCC 33047 was studied under nitrogen fixing condition under two different light intensities. It was observed that the organism showed the shortest doubling time of 3.8 ± 0.4 h^-1^ when grown under a light of 1500 μmol photons m^-1^s^-1^. This is one of the shortest reported for diazotrophic cyanobacteria under N_2_-fixing conditions.
