# Supplementary material for: A Genome-Scale Metabolic Model of Anabaena 33047 to Guide Genetic Modifications to Overproduce Nylon Monomers
Source: Metabolites. 2021 Mar 15;11(3):168. doi: 10.3390/metabo11030168 (PMC7999273; doi:10.3390/metabo11030168)
Supplement: Supplementary file 1 [file metabolites-11-00168-s001.zip › supp_info/Additional File S2.pdf]

Two_cell_model_reduction_level_10_merge_ex

Expand All Readme

Independent Section

Contains tests that are independent of the class of modeled organism, a model's complexity or types of identifiers that are used to describe its components. Parameterization or initialization of the network is not required. See readme for more details.

Consistency

Stoichiometric Consistency	99.1%
Mass Balance	97.4%
Charge Balance	100.0%
Metabolite Connectivity	100.0%
Unbounded Flux In Default Medium	100.0%
Sub Total	99%

Annotation - Metabolites

Presence of Metabolite Annotation	100.0%
**Metabolite Annotations Per Database**	Info
pubchem.compound	0.0%
kegg.compound	0.0%
seed.compound	96.4%
inchikey	0.0%
inchi	0.0%
chebi	0.0%
hmdb	0.0%
reactome	0.0%
metanetx.chemical	0.0%
bigg.metabolite	0.0%
biocyc	0.0%
**Metabolite Annotation Conformity Per Database**	Info
pubchem.compound	0.0%
kegg.compound	0.0%
seed.compound	100.0%
inchikey	0.0%
inchi	0.0%
chebi	0.0%
hmdb	0.0%
reactome	0.0%
metanetx.chemical	0.0%
bigg.metabolite	0.0%
biocyc	0.0%
Uniform Metabolite Identifier Namespace	0.0%
Sub Total	29%

Annotation - Reactions

Presence of Reaction Annotation	100.0%
**Reaction Annotations Per Database**	Info
rhea	0.0%
kegg.reaction	0.0%
seed.reaction	86.0%
metanetx.reaction	0.0%
bigg.reaction	0.0%
reactome	0.0%
ec-code	0.0%
brenda	0.0%
biocyc	0.0%
**Reaction Annotation Conformity Per Database**	Info
rhea	0.0%
kegg.reaction	0.0%
seed.reaction	100.0%
metanetx.reaction	0.0%
bigg.reaction	0.0%
reactome	0.0%
ec-code	0.0%
brenda	0.0%
biocyc	0.0%
Uniform Reaction Identifier Namespace	97.6%
Sub Total	55%

Annotation - Genes

Presence of Gene Annotation	100.0%
**Gene Annotations Per Database**	Info
refseq	0.0%
uniprot	0.0%
ecogene	0.0%
kegg.genes	0.0%
ncbigi	0.0%
ncbigene	0.0%
ncbiprotein	100.0%
ccds	0.0%
hprd	0.0%
asap	0.0%
**Gene Annotation Conformity Per Database**	Info
refseq	0.0%
uniprot	0.0%
ecogene	0.0%
kegg.genes	0.0%
ncbigi	0.0%
ncbigene	0.0%
ncbiprotein	98.0%
ccds	0.0%
hprd	0.0%
asap	0.0%
Sub Total	40%

Annotation - SBO Terms

Metabolite General SBO Presence	100.0%
Metabolite SBO:0000247 Presence	100.0%
Reaction General SBO Presence	100.0%
Metabolic Reaction SBO:0000176 Presence	100.0%
Transport Reaction SBO:0000185 Presence	88.6%
Exchange Reaction SBO:0000627 Presence	100.0%
Demand Reaction SBO:0000628 Presence	100.0%
Sink Reactions SBO:0000632 Presence	Skipped
Gene General SBO Presence	0.0%
Gene SBO:0000243 Presence	0.0%
Biomass Reactions SBO:0000629 Presence	100.0%
Sub Total	72%

Total Score	76%

Total Score

76%

Score per Category
Export

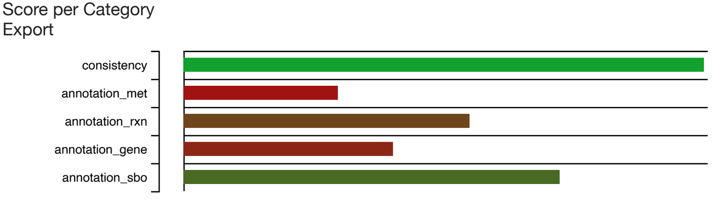

Specific Section

Covers general statistics and specific aspects of a metabolic network that are not universally applicable. See readme for more details.

SBML

SBML Level and Version	Errored
FBC enabled	Errored

Basic Information

Model Identifier	Two_cell_model_reduction_level_10_merge_ex
Total Metabolites	1,811
Total Reactions	1,845
Total Genes	892
Total Compartments	9
Metabolic Coverage	2.07

Metabolite Information

Unique Metabolites	1,811
Duplicate Metabolites in Identical Compartments	0
Metabolites without Charge	0
Metabolites without Formula	0
Medium Components	19

Reaction Information

Purely Metabolic Reactions	1,577
Purely Metabolic Reactions with Constraints	2
Transport Reactions	202
Transport Reactions with Constraints	0
Thermodynamic Reversibility of Purely Metabolic Reactions	1.00
Reactions With Partially Identical Annotations	0.00
Duplicate Reactions	0.00
Reactions With Identical Genes	0.90

Gene-Protein-Reaction (GPR) Associations

Reactions without GPR	131
Fraction of Transport Reactions without GPR	0.54
Enzyme Complexes	150

Biomass

Biomass Reactions Identified	20
**Biomass Consistency**	Info
Cellwall_biomass__hc	0.37
Cellwall_biomass__vc	0.37
DNA_biomass__hc	1.00
DNA_biomass__vc	1.00
Inorganicions_biomass__hc	1.00
Inorganicions_biomass__vc	1.00
Lipid_biomass__hc	0.00
Lipid_biomass__vc	0.00
Pigment_biomass__hc	0.98
Pigment_biomass__vc	0.98
Prot_biomass__hc	1.01
Prot_biomass__vc	1.01
RNA_biomass__hc	1.00
RNA_biomass__vc	1.00
Solpool_biomass__hc	0.28
Solpool_biomass__vc	0.28
biomass_eq_33047__hc	0.12
biomass_eq_33047__vc	0.12
carb_biomass__hc	0.16
carb_biomass__vc	0.16
**Biomass Production In Default Medium**	Info
Cellwall_biomass__hc	0.00
Cellwall_biomass__vc	0.00
DNA_biomass__hc	0.00
DNA_biomass__vc	0.00
Inorganicions_biomass__hc	0.00
Inorganicions_biomass__vc	0.00
Lipid_biomass__hc	0.02
Lipid_biomass__vc	0.02
Pigment_biomass__hc	0.01
Pigment_biomass__vc	0.01
Prot_biomass__hc	0.10
Prot_biomass__vc	0.10
RNA_biomass__hc	0.02
RNA_biomass__vc	0.02
Solpool_biomass__hc	0.01
Solpool_biomass__vc	0.01
biomass_eq_33047__hc	0.24
biomass_eq_33047__vc	0.24
carb_biomass__hc	0.32
carb_biomass__vc	0.32
**Unrealistic Growth Rate In Default Medium**	Info
Cellwall_biomass__hc	false
Cellwall_biomass__vc	false
DNA_biomass__hc	false
DNA_biomass__vc	false
Inorganicions_biomass__hc	false
Inorganicions_biomass__vc	false
Lipid_biomass__hc	false
Lipid_biomass__vc	false
Pigment_biomass__hc	false
Pigment_biomass__vc	false
Prot_biomass__hc	false
Prot_biomass__vc	false
RNA_biomass__hc	false
RNA_biomass__vc	false
Solpool_biomass__hc	false
Solpool_biomass__vc	false
biomass_eq_33047__hc	false
biomass_eq_33047__vc	false
carb_biomass__hc	false
carb_biomass__vc	false
**Biomass Production In Complete Medium**	Info
Cellwall_biomass__hc	0.00
Cellwall_biomass__vc	0.00
DNA_biomass__hc	0.10
DNA_biomass__vc	0.10
Inorganicions_biomass__hc	0.20
Inorganicions_biomass__vc	0.20
Lipid_biomass__hc	2.00
Lipid_biomass__vc	2.00
Pigment_biomass__hc	0.50
Pigment_biomass__vc	0.50
Prot_biomass__hc	9.14
Prot_biomass__vc	9.14
RNA_biomass__hc	1.80
RNA_biomass__vc	1.80
Solpool_biomass__hc	0.57
Solpool_biomass__vc	0.57
biomass_eq_33047__hc	20.94
biomass_eq_33047__vc	20.94
carb_biomass__hc	27.93
carb_biomass__vc	27.93
**Blocked Biomass Precursors In Default Medium**	Info
Cellwall_biomass__hc	Errored
Cellwall_biomass__vc	Errored
DNA_biomass__hc	Errored
DNA_biomass__vc	Errored
Inorganicions_biomass__hc	Errored
Inorganicions_biomass__vc	Errored
Lipid_biomass__hc	Errored
Lipid_biomass__vc	Errored
Pigment_biomass__hc	Errored
Pigment_biomass__vc	Errored
Prot_biomass__hc	Errored
Prot_biomass__vc	Errored
RNA_biomass__hc	Errored
RNA_biomass__vc	Errored
Solpool_biomass__hc	Errored
Solpool_biomass__vc	Errored
biomass_eq_33047__hc	Errored
biomass_eq_33047__vc	Errored
carb_biomass__hc	Errored
carb_biomass__vc	Errored
**Blocked Biomass Precursors In Complete Medium**	Info
Cellwall_biomass__hc	Errored
Cellwall_biomass__vc	Errored
DNA_biomass__hc	Errored
DNA_biomass__vc	Errored
Inorganicions_biomass__hc	Errored
Inorganicions_biomass__vc	Errored
Lipid_biomass__hc	Errored
Lipid_biomass__vc	Errored
Pigment_biomass__hc	Errored
Pigment_biomass__vc	Errored
Prot_biomass__hc	Errored
Prot_biomass__vc	Errored
RNA_biomass__hc	Errored
RNA_biomass__vc	Errored
Solpool_biomass__hc	Errored
Solpool_biomass__vc	Errored
biomass_eq_33047__hc	Errored
biomass_eq_33047__vc	Errored
carb_biomass__hc	Errored
carb_biomass__vc	Errored
**Ratio of Direct Metabolites in Biomass Reaction**	Info
Cellwall_biomass__hc	Errored
Cellwall_biomass__vc	Errored
DNA_biomass__hc	Errored
DNA_biomass__vc	Errored
Inorganicions_biomass__hc	Errored
Inorganicions_biomass__vc	Errored
Lipid_biomass__hc	Errored
Lipid_biomass__vc	Errored
Pigment_biomass__hc	Errored
Pigment_biomass__vc	Errored
Prot_biomass__hc	Errored
Prot_biomass__vc	Errored
RNA_biomass__hc	Errored
RNA_biomass__vc	Errored
Solpool_biomass__hc	Errored
Solpool_biomass__vc	Errored
biomass_eq_33047__hc	Errored
biomass_eq_33047__vc	Errored
carb_biomass__hc	Errored
carb_biomass__vc	Errored
**Number of Missing Essential Biomass Precursors**	Info
Cellwall_biomass__hc	Errored
Cellwall_biomass__vc	Errored
DNA_biomass__hc	Errored
DNA_biomass__vc	Errored
Inorganicions_biomass__hc	Errored
Inorganicions_biomass__vc	Errored
Lipid_biomass__hc	Errored
Lipid_biomass__vc	Errored
Pigment_biomass__hc	Errored
Pigment_biomass__vc	Errored
Prot_biomass__hc	Errored
Prot_biomass__vc	Errored
RNA_biomass__hc	Errored
RNA_biomass__vc	Errored
Solpool_biomass__hc	Errored
Solpool_biomass__vc	Errored
biomass_eq_33047__hc	Errored
biomass_eq_33047__vc	Errored
carb_biomass__hc	Errored
carb_biomass__vc	Errored

Energy Metabolism

Non-Growth Associated Maintenance Reaction	Errored
**Growth-associated Maintenance in Biomass Reaction**	Info
Cellwall_biomass__hc	Errored
Cellwall_biomass__vc	Errored
DNA_biomass__hc	Errored
DNA_biomass__vc	Errored
Inorganicions_biomass__hc	Errored
Inorganicions_biomass__vc	Errored
Lipid_biomass__hc	Errored
Lipid_biomass__vc	Errored
Pigment_biomass__hc	Errored
Pigment_biomass__vc	Errored
Prot_biomass__hc	Errored
Prot_biomass__vc	Errored
RNA_biomass__hc	Errored
RNA_biomass__vc	Errored
Solpool_biomass__hc	Errored
Solpool_biomass__vc	Errored
biomass_eq_33047__hc	Errored
biomass_eq_33047__vc	Errored
carb_biomass__hc	Errored
carb_biomass__vc	Errored
Number of Reversible Oxygen-Containing Reactions	26
**Erroneous Energy-generating Cycles**	Info
MNXM3	Skipped
MNXM63	Skipped
MNXM51	Skipped
MNXM121	Skipped
MNXM423	Skipped
MNXM6	Skipped
MNXM10	Skipped
MNXM38	Skipped
MNXM208	Skipped
MNXM191	Skipped
MNXM223	Skipped
MNXM7517	Skipped
MNXM12233	Skipped
MNXM558	Skipped
MNXM21	Skipped
MNXM89557	Skipped

Network Topology

Universally Blocked Reactions	563
Orphan Metabolites	156
Dead-end Metabolites	149
Stoichiometrically Balanced Cycles	0
Metabolite Production In Complete Medium	654
Metabolite Consumption In Complete Medium	673

Matrix Conditioning

Ratio Min/Max Non-Zero Coefficients	0.00
Independent Conservation Relations	222
Rank	1589
Degrees Of Freedom	256

Experimental Data Comparison

Growth Prediction	Skipped
Gene Essentiality Prediction	Skipped

Misc. Tests

Environment

Python Version	3.6.10
Platform	Linux
Memote Version	0.10.2
