# Supplementary figures and images for: A Genome-Scale Metabolic Model of Anabaena 33047 to Guide Genetic Modifications to Overproduce Nylon Monomers
Source: Metabolites. 2021 Mar 15;11(3):168. doi: 10.3390/metabo11030168 (PMC7999273; doi:10.3390/metabo11030168)

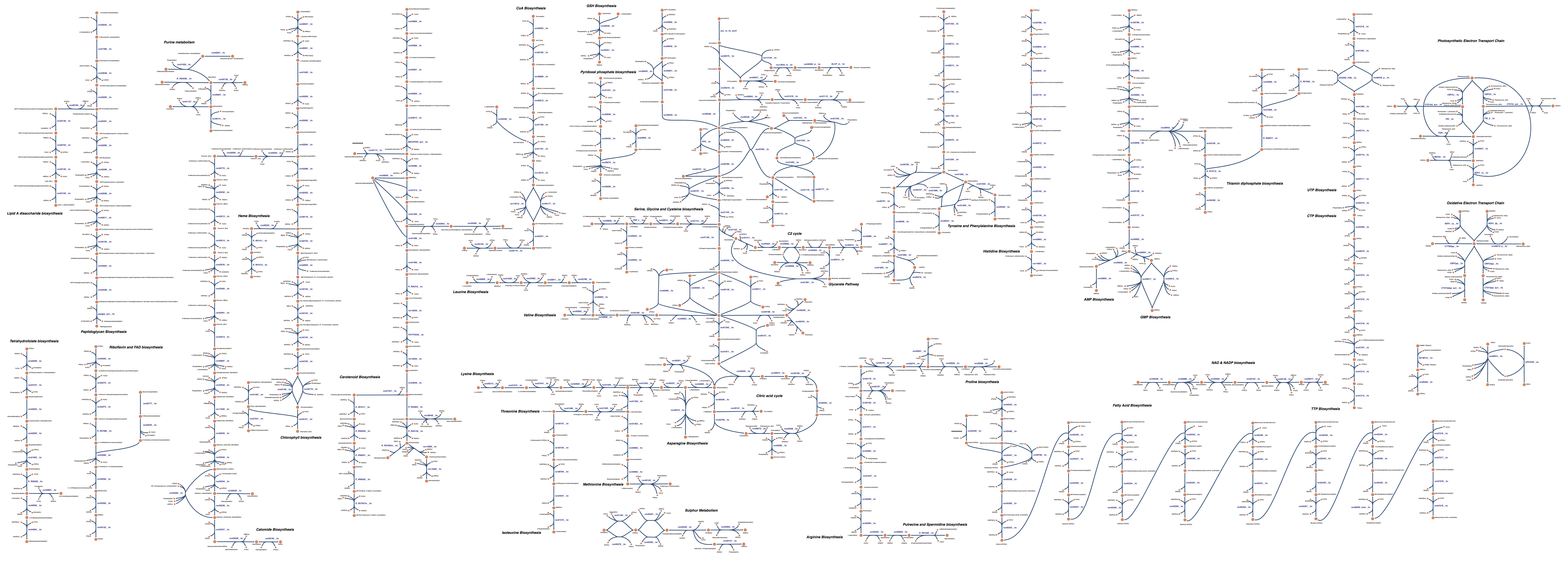

Supplement: Supplementary file 1 [file metabolites-11-00168-s001.zip › supp_info/Figure S2.png]

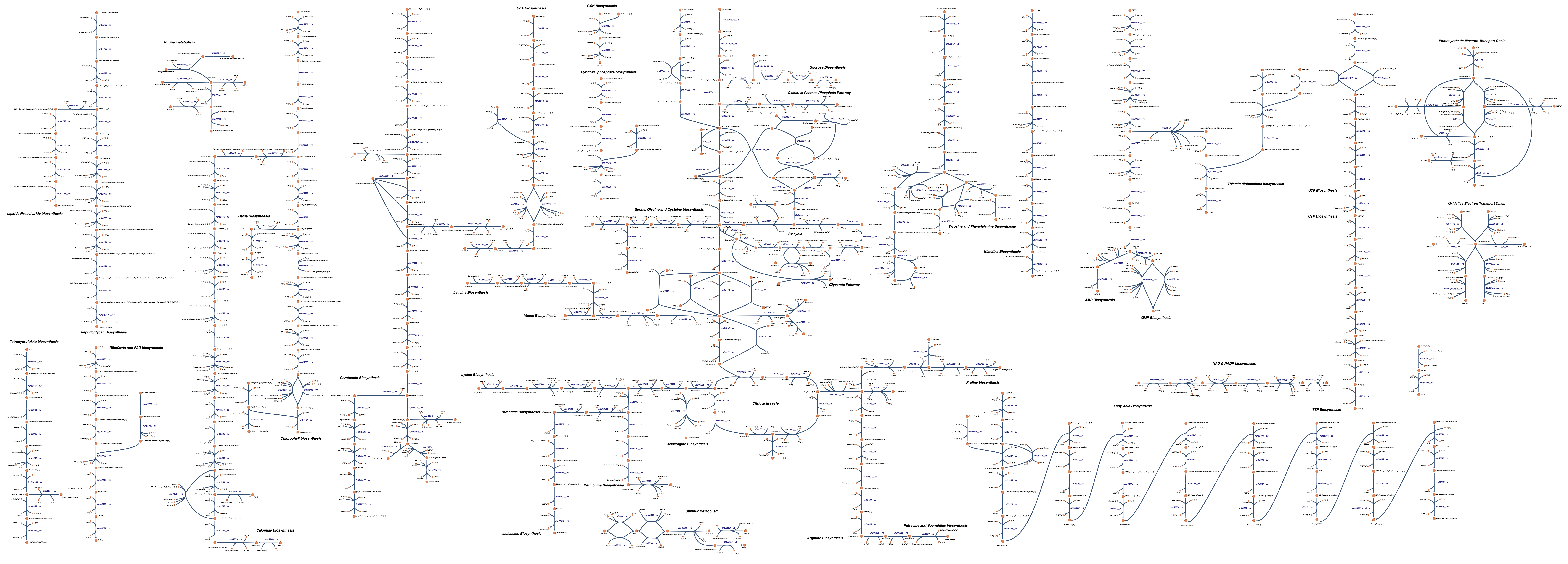

Supplement: Supplementary file 1 [file metabolites-11-00168-s001.zip › supp_info/Figure S1.png]
